# miRNA Profiling of Naïve, Effector and Memory CD8 T Cells

**DOI:** 10.1371/journal.pone.0001020

**Published:** 2007-10-10

**Authors:** Haoquan Wu, Joel R. Neilson, Priti Kumar, Monika Manocha, Premlata Shankar, Phillip A. Sharp, N. Manjunath

**Affiliations:** 1 The CBR Institute for Biomedical Research and Department of Pediatrics, Harvard Medical School, Boston, Massachusetts, United States of America; 2 Center for Cancer Research, Massachusetts Institute of Technology, Cambridge, Massachusetts, United States of America; 3 Department of Biology, Massachusetts Institute of Technology, Cambridge, Massachusetts, United States of America; Columbia University, United States of America

## Abstract

microRNAs have recently emerged as master regulators of gene expression during development and cell differentiation. Although profound changes in gene expression also occur during antigen-induced T cell differentiation, the role of miRNAs in the process is not known. We compared the miRNA expression profiles between antigen-specific naïve, effector and memory CD8+ T cells using 3 different methods-small RNA cloning, miRNA microarray analysis and real-time PCR. Although many miRNAs were expressed in all the T cell subsets, the frequency of 7 miRNAs (miR-16, miR-21, miR-142-3p, miR-142-5p, miR-150, miR-15b and let-7f) alone accounted for ∼60% of all miRNAs, and their expression was several fold higher than the other expressed miRNAs. Global downregulation of miRNAs (including 6/7 dominantly expressed miRNAs) was observed in effector T cells compared to naïve cells and the miRNA expression levels tended to come back up in memory T cells. However, a few miRNAs, notably miR-21 were higher in effector and memory T cells compared to naïve T cells. These results suggest that concomitant with profound changes in gene expression, miRNA profile also changes dynamically during T cell differentiation. Sequence analysis of the cloned mature miRNAs revealed an extensive degree of end polymorphism. While 3′end polymorphisms dominated, heterogeneity at both ends, resembling drosha/dicer processing shift was also seen in miR-142, suggesting a possible novel mechanism to generate new miRNA and/or to diversify miRNA target selection. Overall, our results suggest that dynamic changes in the expression of miRNAs may be important for the regulation of gene expression during antigen-induced T cell differentiation. Our study also suggests possible novel mechanisms for miRNA biogenesis and function.

## Introduction

During an immune response, antigen-specific naïve T cells proliferate enormously and develop into effector T cells capable of immediate effector functions such as cytotoxicity and cytokine production[1], [2]. After clearance of antigen, most of the effector T cells are eliminated by activation-induced cell death, but some antigen experienced cells differentiate into relatively quiescent memory T cells which persist over long periods of time and mount a rapid and augmented response upon rechallenge with the antigen[3], [4], [5]. While naïve and memory T cells are quiescent and express molecules needed for trafficking from blood to lymphoid organs, effector cells are actively dividing, produce various effector cytokines and cytotoxic molecules, and express molecules needed for trafficking into tissue sites of infection[6], [7]. Thus, major changes in gene expression characterize these differentiation states, with gene expression getting enormously upregulated in effector T cells, but decreasing in memory T cells[8]. Although transcriptional regulation of gene expression has been well studied, the role of microRNAs in the process of T cell differentiation is unclear.

RNA interference mediated by microRNAs has emerged as an important mechanism for global regulation of gene expression in plants and animals, including humans (reviewed in ref[9], [10]). miRNAs are small RNAs that regulate gene expression by translational repression and/or mRNA degradation[9], [11]. miRNAs are genomically encoded and are transcribed in the nucleus as long primary miRNAs, which are then processed by drosha-DGCR8 into ∼70 bp pre miRNA[12], [13], [14]. The pre-miRNA is exported out of the nucleus by exportin 5[15], [16] and is further processed in the cytoplasm by the enzyme dicer into ∼22 nt double stranded mature miRNA[17], [18], [19]. One strand of the miRNA associates with RNA induced silencing complex (RISC) to mediate gene silencing[20]. Generally the miRNA is imperfectly complementary to the 3′UTR sequence of the target mRNA and forms a bulge upon binding[21]. The 5′ 2–7 nt of the miRNA strand is perfectly complementary (seed region) and defines the target specificity of the miRNA[22], [23], [24]. A single miRNA can regulate many target mRNAs containing binding sites for that miRNA [25].

A number of miRNA profiling studies have shown that the expression of miRNA changes during hematopoietic development and carcinogenesis, suggesting that miRNAs might be important players in these processes[26], [27], [28], [29]. Although the exact functional consequence of expression of a given miRNA is largely unknown, recent studies have clearly demonstrated the importance of individual miRNAs in hematopoietic cell development and function. For instance, miR-181a has been shown to regulate T and B cell development when ectopically expressed in hematopoietic progenitor cells[26]. A more recent paper has further demonstrated that miR-181a could modulate T cell sensitivity to antigen stimulation[30]. Another study showed that miR-150 could block B cell development when expressed prematurely[31]. Similarly, miR-155 has been shown to regulate germinal center reaction as well as helper T cell response[32], [33]. Although these studies suggest that miRNAs have important roles in immune cell development and function, a comprehensive analysis of miRNA expression during antigen-specific T cell differentiation is still lacking. Thus in this study, we analyzed the miRNA expression profile in antigen-specific naïve, effector and memory CD8 T cells using the method we have described earlier to derive these cell types[34]. We report that a few miRNAs are dominantly expressed and that their expression pattern changes during the differentiation process. We also note a high degree of heterogeneity in the mature miRNA ends in the T cell subsets.

## Results

### Dynamic regulation of miRNAs during antigen-induced CD8 T cell differentiation

**Figure 1:**
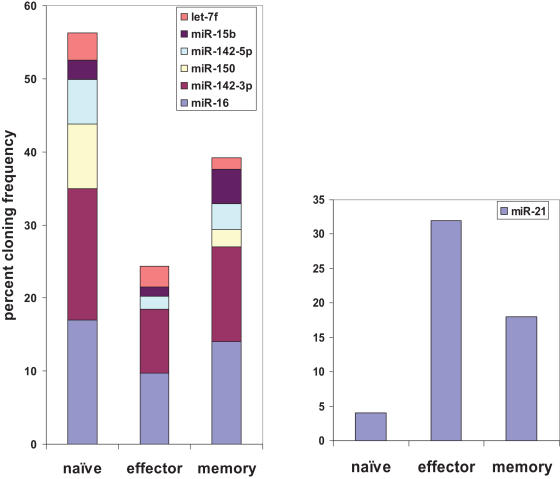
Distribution of the seven highly expressed miRNAs in the T cell subsets. The cloning frequency of each miRNA is shown as a percentage of total miRNA clones isolated for each T cell subset.

To determine if the expression profile of microRNAs changes during T cell differentiation, small RNAs were cloned and sequenced from pure populations of antigen-specific naïve, effector and memory CD8 T cells, generated as described earlier[34]. In total, 1498 small RNAs were cloned and their length ranged from 15 to 35 nucleotides, with a peak at 21 to 23 nucleotides. The cloned sequences were queried using BLASTN against miRBase (v.10.0). With respect to the annotated sequence of a particular miRNA, one single nucleotide internal mismatch and up to 3 nucleotide mismatch at the 3′ end were allowed to designate the sequence as the miRNA. Up to 3 nucleotide addition or deletion at both ends were also allowed, as long as these nucleotides completely matched the miRNA genomic sequence. Of 1498 cloned sequences, 837 (343 in naïve, 240 in effector and 254 in memory T cells) clones corresponded to previously annotated miRNAs. Many of the miRNAs were cloned multiple times and the total number of individual microRNAs cloned in naïve, effector and memory T cells constituted 69, 51 and 54 respectively. The distribution of various microRNAs, the number of clones isolated for each miRNA (miRNA frequency) as well as their representation as a percent of total miRNA pool for each of the different T cell subsets are depicted in Supplemental Table S1. Although a total of 94 known miRNAs were cloned at varying frequencies, some miRNAs dominated in all subsets. Strikingly, clones from seven miRNAs (miR-16, miR-21, miR-142-3p, miR-142-5p, miR-150, miR-15b and let-7f) constituted nearly 60% of the total clones and of these, 3 miRNAs (miR-16, miR-142-3p and miR-21) alone accounted for ∼40% of all clones (Table S1 and Fig 1), suggesting that these are the dominantly expressed miRNAs in CD8 T cells.

Among the 7 highest expressed miRNAs, 6 showed dramatically reduced frequencies in effector T cells compared to naïve T cells, and the expression of these miRNAs tended to increase back in memory T cells (Fig. 1, left panel). However miR-21 was an exception, showing reverse kinetics with highest frequency in effector T cells followed by memory T cells and lowest expression in naïve T cells (Fig. 1, right panel).

**Figure 2:**
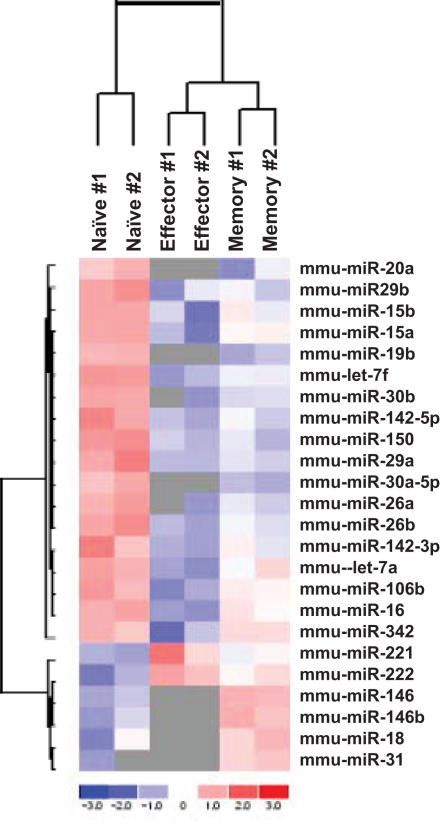
Differential expression of miRNAs in naïve, effector and memory T cells. Signal intensity for a particular miRNA in each cell type was divided by that of the pooled RNA reference sample to generate the heat map. Same amount of total RNA is used for each slide (so the signal is actually normalized according to total RNA). The miRNA clustering tree is shown on the left and the sample clustering tree is shown on the top. The color scale shown at the bottom illustrates the relative expression level of the indicated miRNA across all samples: red denotes an expression above the mean, blue denotes an expression lower than the mean and grey represents signal lower than the background.

Although direct cloning has been widely used to profile the miRNA expression level, it may not be a good method for the relative quantitation of low frequency miRNAs, particularly when small-scale sequencing is used as done in this study. Moreover, although it is a good method to determine the relative expression level of miRNAs within a given sample, it may not be ideal for comparison of expression levels between different samples because it is hard to normalize the data between samples. Therefore, we also used a miRNA microarray to further analyze the expression profiles in the different T cell subsets. RNAs from T cell subsets generated in 2 independent experiments were profiled with LNA (locked nucleotide acid)-based miRNA microarray. Essentially similar results were seen in both sets attesting to the reproducibility of the results (Fig. 2).

Analysis of the expression levels in the microarray confirmed that the 6 (out of 7) miRNAs (miR-16, miR-142-3p, miR-142-5p, miR-150, miR-15b and let-7f) that showed the highest frequency in direct cloning, were significantly down regulated in effector cells compared to naïve T cells and the expression tended to increase back in memory T cells (Fig 2) as observed in direct cloning method. Moreover, this pattern of differences was also evident for most of the low frequency miRNAs—around 80% of miRNAs had the same pattern (Fig 2 and Table S2). In fact, although the same amount of total RNA was used for each sample (so that all the microarray data are actually normalized to total RNA amount), the total miRNA hybridization signal in naïve T cells was 2.98 fold higher than in effector T cells and 1.45 fold higher than in memory T cells (Table S2), suggesting that the total amount of miRNAs in effector T cells is only ∼33% of that in naïve T cells, and the miRNA expression increased in memory T cells to constitute ∼69% of that in naïve T cells.

**Figure 3:**
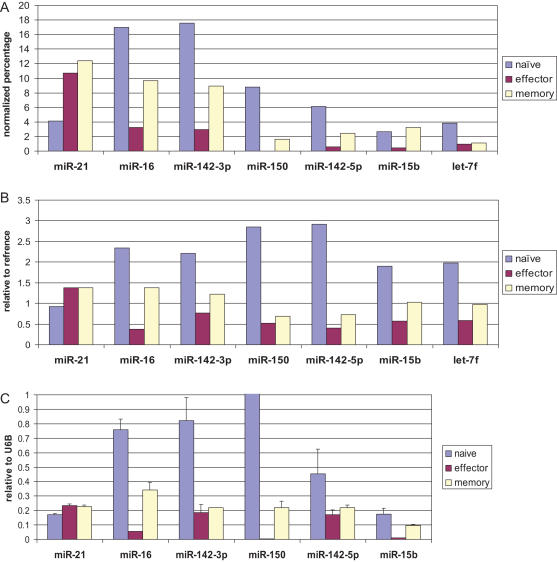
Comparison of miRNA expression profile in the T cell subsets by 3 different methods. A) Expression profile by direct cloning. The cloning frequency was normalized to the total hybridization signal in the miRNA microarray. B) Expression profile by microarray hybridization. Ratios of sample/reference hybridization signal is shown. C) Expression profile by real time PCR. Expression level was normalized to that of small non-coding RNA U6B. Mean of triplicate experiments±SD is shown. Similar results were also obtained in 2 additional independent experiments.

Because it is hard to normalize the data between samples in direct cloning, to more rigorously reanalyze the direct cloning data, the cloning data was normalized according to total hybridization signal from the array (by dividing the cloning frequency in effector and memory cells by 2.98 and 1.45 respectively, Table S3). When examined in this way, the expression level in both assays showed a good degree of correlation (Fig 3 A, B). Taken together, our results suggest that except for a few miRNAs such as miR-21 and miR-31, most miRNAs are down regulated in effector T cells compared to naïve T cell and tend to increase back in memory T cells.

**Figure 4:**
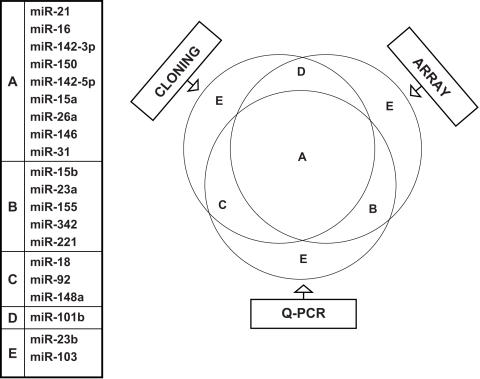
Correlation amongst three methods of miRNA expression profiling. Twenty different miRNAs (indicated on the left) that were picked up by direct cloning and/or microarray hybridization were also tested by real time PCR. A shows miRNAs whose relative expression between naïve, effector and memory T cells correlated in all 3 methods. B shows miRNAs with expression profile correlated only in the microarray and real time RT-PCR. C shows the expression profile that correlated only in cloning and RT-PCR. D shows the expression profile that correlated only in cloning and microarray and E represents the miRNAs that did not correlate in any two methods. The correlation was determined arbitrarily according to their movement in the same direction in the different assays.

We also confirmed the T cell subset-specific differences in miRNA expression in a real-time PCR assay. Because the extensive 3′ end heterogeneity that we noted in miRNAs cloned from T cells (discussed later) might affect detection by ABI Taqman microRNA assay, we used a 3′RACE RT-PCR based real-time PCR. The PCR results for the 6 highly expressed miRNAs correlated well with both the array data and the normalized cloning data (Fig 3 A, B and C). However, examination of let-7f (one of the 7 highest expressed miRNAs) was excluded in this assay because of the difficulty in designing primers that can distinguish the different let-7 family members without ambiguity. We also performed real-time PCR for another 14 miRNAs that were expressed at lower levels (Fig S1). We arbitrarily determined the correlation according to their movement in the same direction among the 3 methods. The venn diagram (Fig 4) depicts the extent of correlation among the 3 methods for all 20 miRNAs tested by real-time PCR. Differential expression of 9 miRNAs in naïve, effector and memory T cells could be confirmed by all 3 methods (overlap in the diagram). The expression levels of 9 other miRNAs correlated in 2 assays and only 2 miRNAs did not correlate in any 2 methods. Overall, our results clearly show that miRNA expression is dynamically regulated during antigen-specific T cell differentiation.

Changes in miRNA expression such as downregulation of miR-150 and upregulation of miR-155 has been reported previously following a brief stimulation of T cells with αCD3[35], [36]. However, we found a striking global downregulation of miRNA in antigen stimulated T cell that had fully differentiated into effector T cells, seven days after stimulation. It is of interest to further examine the temporal pattern of miRNA changes at different times after antigen stimulation.

### Identification of a novel miRNA—miR669d in T cells

**Figure 5:**
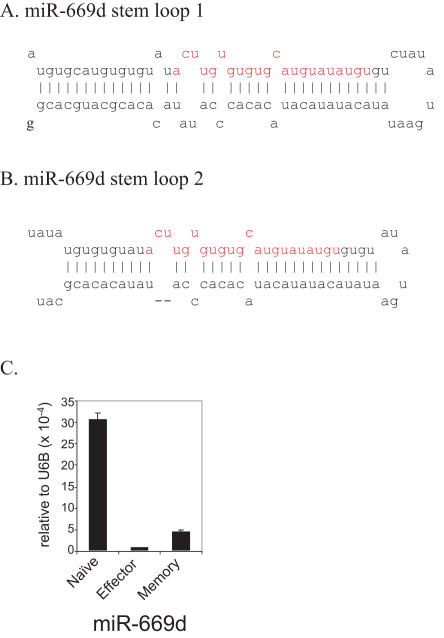
Novel miRNA–miR-669d in mouse T cells. The presence of the stem loop sequences for miRNA-669d in the mouse genome is depicted in (A-B). C) Small RNAs from mouse T cells were tested for the presence of miR-669d by real time RT-PCR. Expression level was normalized to that of small non-coding RNA U6B. Mean of triplicate experiments±SD is shown.

We also cloned what appears to be a novel miRNA, not annotated in the latest version of miRBase (v.10.0). A sequence (ACUUGUGUGUGCAUGUAUAUGU) that appears to be a homologue of miR-699a (A***G***UUGUGUGUGCAUGU***UC***AUGU) was cloned once in both naïve and memory T cells. Interestingly, this sequence was found to perfectly match two regions in the of murine chromosome 2: nt 10386237-10386258(+) and nt 10389511-10389532(+), which is located in intron 10 of gene sfmbt2 (Scm-like with four mbt domains 2). The sequence forms a typical stem loop structure that appears to be the precursor of miRNA (Fig. 5 A, B). Its expression in murine T cells was also confirmed by real-time PCR (Fig. 5 C). Therefore, we have designated the novel miRNA as mmu-miR-669d.

### Heterogeneity in the mature microRNA sequences

A detailed analysis of the cloning data revealed that compared to the mature miRNA sequences annotated in the miRBase, the miRNAs in T cells exhibit an extensive degree of polymorphism at the ends. Although some clones in each miRNA contained the exact match for the annotated mature miRNA sequence, the majority of the clones within a given miRNA did not match the sequence in the miRBase (release 10.0). For example, the most prevalent sequence for miR-21 we found was UAGCUUAUCAGACUGAUGUUGA***C***, while in the miRBase it is UAGCUUAUCAGACUGAUGUUGA. Similarly, the prevalent sequence for miR-142-3p was GUAGUGUUUCCUACUUUAUGGA while in the miRBase it is ***U***GUAGUGUUUCCUACUUUAUGGA and for miR-142-5p, it was ***CC***CAUAAAGUAGAAAGCACUAC in our study, while in the miRBase it is CAUAAAGUAGAAAGCACUAC***U***. Also the most prevalent sequence varied between different T cell subsets. For example, the most prevalent sequence for miR-21 in naïve T cells was UAGCUUAUCAGACUGAUGUUGA (57% of total), while in effector and memory cells it is UAGCUUAUCAGACUGAUGUUGA***C*** (64% of total, Table 1).

**Table 1:** Examples of miRNA end variations in the T cell subsets.

		Sequence	Reads		
			Naive	Effector	Memory
**miR-21**	template	ATAGCTTATCAGACTGATGTTGACTGT			
	templated	..TAGCTTATCAGACTGATGTTGACT…..	0	2	0
		..TAGCTTATCAGACTGATGTTGAC……	2	47	26
		..TAGCTTATCAGACTGATGTTGA……..	8	18	17
		..TAGCTTATCAGACTGATGTTG……….	4	0	0
		……..CTTATCAGACTGATGTTGA……..			1
		….…....TTATCAGACTGATGTTGAC……		2	
		…….....TTATCAGACTGATGTTGA……...		1	
	untemplated	..TAGCTTATCAGACTGATGTTGACc….		1	1
		..TAGCTTATCAGACTGATGTTGACa….		1	
		..TAGCTTATCAGACTGATGTTGACg….		1	
		gTAGCTTATCAGACTGATGTTGAC……		1	
		gTAGCTTATCAGACTGATGTTGA……..		1	
**miR-16-1**	template	TTAGCAGCACGTAAATATTGGCGTTA			
**miR-16-2**	template	CTAGCAGCACGTAAATATTGGCGTAG			
	templated	.TTAGCAGCACGTAAATATTGGC………	1		
		...TAGCAGCACGTAAATATTGGCGTA...			1
		...TAGCAGCACGTAAATATTGGCGT…..	15	5	5
		...TAGCAGCACGTAAATATTGGCG…….	26	15	23
		...TAGCAGCACGTAAATATTGGC………	10	2	4
		….AGCAGCACGTAAATATTGGCGT…..		1	
	untemplated	...TAGCGGCACGTAAATATTGGCGT…..			1
		...TAGCAGCACGTAAATATTGGCGa…….	5		
		...TAGCAGCACGTAAATATTGGCaa…….			1
		...TAGCAGCACGTAAATATTGGCa…….			1
		...TAGCAGCACGTAAATATTGGaa.…….	1		
**miR-142-3p**	template	GTGTAGTGTTTCCTACTTTATGGATGT			
	templated	...TGTAGTGTTTCCTACTTTATGGA……	5	3	2
		...TGTAGTGTTTCCTACTTTATGG..……	6	3	8
		...TGTAGTGTTTCCTACTTTAT…………	3	0	0
		….GTAGTGTTTCCTACTTTATGGA……	18	11	16
		….GTAGTGTTTCCTACTTTATGG..……	11	1	3
		……TAGTGTTTCCTACTTTATGGAT.…	1		
		……TAGTGTTTCCTACTTTATGGA……	1		1
		……..AGTGTTTCCTACTTTATGGATGT	1		
		……..AGTGTTTCCTACTTTATGGA……	1		
		……..AGTGTTTCCTACTTTATGG..……	1		
		…………..GTTTCCTACTTTATGGA……	1		
	untemplated	...TGTAGTGTTTCCTACTTTATGGtt..….			1
		...TGTAGTGTTTCCTACTTTATGGt..……			1
		...TGTAGTGTTTCCTACTTTATGGAa..…	1		
		...TGTAGTGTTTCCTACTTTATGa...……	1		
		….GTAGTGTTTCCTACTTTATGGAaa…	1	1	
		….GTAGTGTTTCCTACTTTATGGAa..…	4	2	1
		….GTAGTGTTTCCTACTTTATGGt….…	2		
		….GTAGTGTTTCCTACTTTATGGg……	1		
		….GTAGTGTTTCCTACTTTATGt…....…	1		
**miR-142-5p**	template	ACCCATAAAGTAGAAAGCACTACTAAC			
	templated	ACCCATAAAGTAGAAAGCACTA…...…..	1		
		ACCCATAAAGTAGAAAGCA……..….….	1		
		..CCCATAAAGTAGAAAGCACTAC……..	5		7
		..CCCATAAAGTAGAAAGCACTA…...…..	3		
		..CCCATAAAGTAGAAAGCACT………...	3		1
		……CATAAAGTAGAAAGCACTACTA….	1		
		……CATAAAGTAGAAAGCACTACT..….	2		
		……CATAAAGTAGAAAGCACTAC….….	1		
	untemplated	..CCCATAAAGTAGAAAGCACTACc...…...		1	
		..CCCATAAAGTAGAAAGCACTACa....…...		1	
		..CCCATAAAGTAGAAAGCACTAt.....…...	2		1
		……CATAAAGTgGAAAGCACTACT..….	1		
		……CATAAAGTAGAAAGCACTgCT..….		1	
		……CATAAAGTAGAAAGCACTACTAt…..		1	
		……CATAAAGTAGAAAGCACTAt..….….	1		

The annotated mature miRNA sequence from miRBase is underlined and a few nucleotides flanking the sequence are also shown in depicting the template. The untemplated nucleotide addition in the cloned sequence is shown in small case letters. A to I editing (A to G conversion) is also shown in small case letters.

Overall, most of the mature miRNAs that were cloned in high frequency had more than 3 variations, with the extreme example of miR-142-3p, which had 20 variations (Tables 1 and S4). The polymorphisms included: 3′ end deletion (∼36% of total); 3′ end extensions (∼15% of total, with preferential incorporation of A or U residues), majority of which were untemplated; and 5′ end variation (∼4% of total, Tables 1 and S4). Such variations have also been noted previously [29], [37], [38]. However, the polymorphism that we observed appears to be more dramatic than previously reported in *C. elegans*: we observed 3′ end heterogeneity in 51% of the 834 annotated miRNA sequences cloned in our study (Table S4) as opposed to the 15% reported for *C. elegans* [38]. Similarly, we found 4% heterogeneity at the 5′ end (Table S4) compared to 0.5% reported in *C. elegans* [38]. And for most miRNAs cloned with high frequency, we found two or more dominant variants (Tables 1 and S4), while the majority of the miRNAs contain a single dominant sequence in *C. elegans* [38].

Compared to 3′ ends, the 5′ ends were more homogenous. However, we did observe variations in 4% of total clones at the 5′ end. An interesting 5′ end variation that we observed appeared to be 5′ end degradation product—3 or more nucleotides were deleted in the 5′ end while the 3′ end remained intact (examples can be seen in miR-21, miR-15b, miR-26a and let-7f in Table S4). This may imply that degradation of these mature miRNAs starts from the 5′ end, which might be an efficient way to eliminate miRNA activity quickly, since the 5′ end is the seed sequence that determines target gene selection[24].

Of particular interest was the variations observed in miR-142, which appeared to be the result of a shift in drosha/dicer processing: 1, 2 or 3 nucleotide deletions in the 5′ end associated with a corresponding increase in the number of templated nucleotides at the 3′ end. This pattern of variation was seen in both miR-142-5p and miR-142-3p at a high frequency (Table 1). Since changes at the 5′ seed sequence (nt 2–7) is thought to affect the miRNA target specificity[39], a shift in drosha/dicer processing might represent a novel mechanism to generate new miRNAs.

Taken together, our results suggest that a given miRNA hairpin may generate more than one product, and therefore designation of a particular sequence for a given mature miRNA may not sufficiently describe all the miRNA forms present inside the cell. Although the relative biological significance of each species within a given miRNA remains to be determined, such variations might possibly affect the stability/subcellular localization, functional efficacy or miRNA target-specificity (particularly with changes in the 5′ end affecting the miRNA “seed” region). However, we cannot rule out the possibility that some of the variations noted may represent cloning artifacts, although this appears unlikely (see Discussion)

### miRNA polymorphism can affect detection by real-time PCR employing stem-loop primer mediated RT-PCR

**Figure 6:**
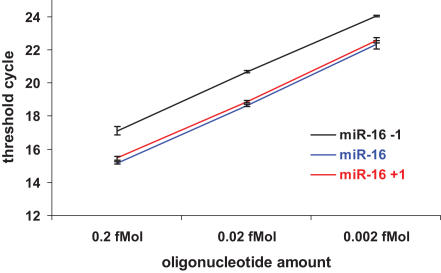
miRNA 3′ end heterogeneity affects detection efficiency of stem-loop primer-based real time PCR. Detection efficiency was compared using exact miR-16 oligonucleotide and miR-16 oligonucleotide with 1 nt deletion or 1 nt addition at the 3′ end as templates. Mean of triplicate experiments±SD is shown.

As mentioned earlier, the miRNA variation at the 3′ end appears to be more frequent than previously thought. This may affect determination of miRNA expression by some assays that depend on the exact match of the 3′ terminus as the basis for high efficiency detection. The real-time PCR employing stem-loop primers that match the 3′ end of miRNA to improve the reverse transcription efficiency is one of the most popular assays to profile and detect miRNA expression[40]. To test if the 3′ end variation affects the detection efficacy, we used miR-16, miR-16+1(one nucleotide extension at 3′ end) and miR-16-1 (one nucleotide deletion at 3′ end) oligonucleotides as templates in the stem-loop primer-based real time PCR developed by ABI (Applied Biosystems, US). Since the exact match of 3′ terminal of miRNA is the basis for high efficiency of this method, 3′end variations should negatively affect detection by this method. Indeed, the efficacy of stem-loop based real time PCR was decreased by 2 cycles in one nucleotide deletion variation, showing that it is only 25% efficient compared to the correctly matched template. Surprisingly however, the efficacy did not change with one nucleotide template extension (Fig. 6).

## Discussion

This is the first report that comprehensively analyzes the miRNA expression profile during antigen-specific T cell differentiation. Our results indicate that only a few miRNAs are dominantly expressed in CD8 T cells and that their expression is dynamically regulated during the differentiation process. Moreover, the level of miRNA expression appears to inversely correlate with the activation status of the cells. For example the level of miRNA expression was highest in the non-replicating and functionally quiescent naïve T cells, but was dramatically reduced in the actively dividing and functionally competent effector T cells and their expression once again increased when the antigen-stimulated T cells were converted into the relatively quiescent memory T cells. Theses results suggest that miRNAs may modulate the differentiation status of antigen-stimulated T cells.

Recent studies in conditional dicer-deficient mice and mice deficient for individual miRNAs have highlighted the importance of miRNAs in regulating T cell development, homeostasis and function[32], [41]. However, a comprehensive study of miRNA profiles during antigen-specific T cell differentiation has not been reported. Our direct cloning results show that although over 90 known miRNAs were cloned from CD8 T cells, only 7 miRNAs were dominantly expressed and constituted ∼60% of the total miRNA pool. This is also consistent with previous studies showing that some miRNAs are dominantly expressed in specific cell types and tissues [29], [37], [42]. Since the miRNA-mediated repression of gene expression is dependent on the miRNA amounts as well as the target mRNA levels[24], the dominantly expressed miRNAs may control the abundantly expressed genes in CD8 T cells. However, we are not suggesting that the other miRNAs expressed at low frequencies are functionally unimportant.

A recent study has shown that regulation of miRNA expression characterizes the stage-specific development of thymocytes[29]. We also found that the miRNA expression levels dynamically change between naïve, effector and memory T cell subsets. A striking global downregulation of miRNA was seen in effector T cells. This trend was evident in all 3 methods examined: direct cloning, microarray profiling as well as RT-PCR. According to total microarray hybridization signal, the total miRNA level is down regulated 2.98 fold in effector compared to naive T cells. Interestingly, the changes in miRNA expression we observed appear to inversely correlate with the degree of cellular activation. Antigen stimulation of naïve T cells results in a massive upregulation of gene expression that is needed for effector T cells to divide actively, gain functional competence and change migratory properties[6], [8], and effector T cell is the cell type where we found the least overall miRNA. Since the upregulation of gene expression occurs at both the protein and mRNA levels, while the miRNA-mediated effect is largely thought to be mediated by translational repression, it is possible that the abundantly expressed miRNAs may primarily control the key transcription factors involved in T cell activation. Massive downregulation of miRNA expression has also been reported in cancer cells [43], [44]. Although the global decrease in miRNA level has been hypothesized to reflect the dedifferentiated status of cancer cells [44], our results suggest that it may indeed reflect the quantum of protein synthesis needed for cellular activity because effector cells are more differentiated compared to naïve T cells. In contrast to the general trend for downregulation in effector T cells, a few miRNAs were higher in effector T cells compared to naïve T cells. Indeed several genes, including those associated with lymph node trafficking, expressed in naïve T cells are silenced in effector T cells[7], [8]. Further functional studies are needed to test if miRNAs upregulated in effector T cells suppress the expression of naïve T cell-specific genes in effector cells.

Two recent studies have also profiled miRNA in CD8 T cells[29], [45] and our results are consistent with both the studies. For example, cloning miRNAs from single positive thymic CD8 T cells, Nelson et al[29] also found that a few miRNAs prevailed in frequency. In fact, the highly expressed miRNAs were also similar to this study, in that 7 miRNAs appeared in both studies among the 10 highest expressed miRNAs, with miR-142-3p showing the highest frequency in both. However, compared to their study, miR-181a and miR-24 were lower in frequency and miR-29a and miR-142-5p were higher in our study. Landgraf et al [45] profiling miRNA expression in human peripheral blood CD8 T cells also found miR-142 to be the highest expressed miRNA. Similarly, they also found high level expression of miR-15a family (including miR-15a, miR-15b and miR-16), miR-21, miR-98 family (including all the let-7), miR-150 and miR-26a.

Although many methods can be used to profile miRNA expression, direct small RNA cloning is the only method that provides actual visualization of mature miRNAs. In our study, direct cloning revealed that mature miRNAs exhibit an extensive degree of end polymorphism in mouse T cells. Although we can not rule out the possibility that some of the observed polymorphism may reflect artifacts of the cloning procedure, this appears to be unlikely because many variant sequences were cloned multiple times and a high frequency of 5′ end variation was only seen in miR-142, while the majority of miRNAs had identical 5′ end. Moreover, although not described in detail except for a study by Ruby et al in C *elegans* [38], miRNA end polymorphism has also been observed by others using small RNA cloning approach[29], [37] and also other methods like RNA-primed Array-based Klenow Extension (RAKE)[46]. Our data show that the ends of mature miRNAs are much more polymorphic than reported in C. *elegans*. We found 2 or more main variations for most mature miRNAs in the mouse T cells while one dominant sequence characterizes most mature miRNAs in *C. elegans*. Although not alluded to by the authors, a careful examination of northern blot results of some published reports also reveal multiple bands for one miRNA[26], [31]. One convincing example in C. *elegans* is miR-84. As shown in Fig. 3 in the paper by Lau and coworkers[47], miR-84 is the only miRNA (out of 15 that were tested) that shows 3 clearly visible bands in northern blots, indicating that miR-84 has 3 main variations. Similarly, an examination of the deep sequencing data depicted in Table S1 in the report by Ruby and coworkers [38] also shows that miR-84 has 3 main variations. Thus, some miRNAs in *C. elegans* also have more than 1 dominant variation, suggesting that multiple dominant sequences for one miRNA is a conserved phenomenon, although it happens to a lower extent in *C. elegans*.

Clearly, the changes in the 5′ end seed sequence can affect target selection[39]. However, the biological significance of the 3′ end polymorphism we observed is not immediately apparent, although the extent of their occurrence suggests that it could be important in miRNA biology. For example, it may possibly affect the stability/subcellular localization and/or miRNA functional efficacy. It has been reported in plants that untemplated 3′ end extension appears to be a signal for mature miRNA degradation[48]. It is possible that it might be the same in mammals. Whatever the significance, our study shows that designation of one sequence for a give miRNA may not be accurate to describe all miRNA species in a cell. Moreover as we have shown, the 3′ end variation can affect the detection efficiency of real-time PCR assay employing stem-loop primer and thus should be taken into consideration when designing a detection method for miRNAs. The method RAKE that requires the exact match of the 3′ end also appears to be affected [46], [49]. Additionally, the real picture of mature miRNA sequence might help generate more accurate models in bioinformatic calculations such as target prediction.

It is intriguing how the end polymorphism is generated. Since a majority of mature miRNAs have identical 5′ ends, irrespective of whether they are derived from the 5′ or 3′ chains of the hairpin, it appears that both drosha and dicer can set a precise 5′ end. Thus, the majority of end polymorphisms is likely generated downstream of drosha/dicer processing. It is reasonable to predict that there are enzymes with end modification activity that could generate mature miRNA end polymorphism.

End polymorphism in miR-142 was very different compared to other miRNAs—both miR-142-5p and miR-142-3p were the only miRNAs that showed a high frequency of 5′ end changes. This polymorphism appears to result from a shift in the drosha/dicer processing, in that 1-3 nucleotide deletions at the 5′ end were associated with a corresponding number of templated nucleotide extensions at the 3′ end. It has recently been proposed that the distance from the stem-flanking ssRNA junction determines the drosha cleavage site and the cleavage occurs approximately 11bp from the junction. Dicer then measures from the terminal set by drosha for further processing[14]. Although most miRNA 5′ ends were intact, suggesting that drosha/dicer processing is precise, the changes observed in miR-142 suggest that a shift in drosha/dicer processing can sometimes occur at a relatively high frequency. Since 5′ end changes can alter target specificity[39], this may also represent a novel mechanism to generate additional miRNAs. Further studies are needed to verify this hypothesis. Although how a shift in drosha/dicer processing occurs is not known, A to I editing may be involved. It has been reported that pri-miR-142 is under heavy A to I editing by ADAR. miR-142 expression level increased significantly after ADAR was mutated and this change appears to be miR-142-specific and not a result of a general effect on the level of miRNA in ADAR mutant mice[50]. Thus, it is possible that the A to I editing may change the stem loop structure or affect the ability of drosha-DGCR8 to cleave precisely. Interestingly, 2 (out of 34) instances of A to I editing could be seen in miR-142-5p, while it was barely detected in other miRNAs in this study (Tables 1 and S4).

In summary, our study documents the changes in miRNA expression during T cell differentiation and points to the extensive degree of polymorphism in mature miRNA ends. Further studies on the differentially expressed miRNAs should aid in the understanding of T cell differentiation process and may ultimately pave the way to manipulate the process of adaptive immunity.

## Materials and Methods

### Mice and T cell subsets

TCR-LCMV-P14/TCR transgenic (specific for LCMV gp33-41) mice were purchased from the Taconic Farms, Germantown, NY. Naïve CD8 T cells were purified from splenocytes of mice by negative selection using the murine T cell CD8 subset isolation kit (R&D systems, Minneapolis, MN) according to the manufacturer's instructions. The isolated cells were >90% pure, and 95% of them were CD62L+, CD69- (not shown). The method to generate effector and memory T cell has been described[34] . Briefly, splenocytes from P14 mice were stimulated with 10 µg/ml of gp33-41 peptide (KAVYNFATC, synthesized at BioSource International) for 1 hour and cultured in RPMI supplemented with 10% FBS. Two days later, the cells were washed and cultured in fresh medium supplemented with 20 ng/ml of either rIL-2 or rIL-15 (R&D Systems) and cultured for 7 days. The cells were then harvested and viable T cells were recovered by Histopaque (Sigma) gradient centrifugation. Almost all viable cells after 7 days of culture were CD8+ and those cultured in IL-2 were CD62L-, CD25+ and those cultured in IL-15 were CD62L+, CD25- (not shown).

### Small RNA cloning

Total RNA and small RNA fractions were isolated using Qiagen miRNeasy kit according to the manufacturer's instruction. The cloning method has been described previously[29]. Briefly, small RNAs were ligated to 3′ adenylated linker (5′-rAppCTGGTATCTGTGTAT GGddC-3′) in a buffer without ATP, followed by 5′ linker (5′-ACCACAGAGAAACC GrCrArG-3′) ligation prior to gel purification. The ligated short RNAs were then separated on 15% urea polyacrylamide gels and purified. After reverse transcription and amplification using 5′-GACTAGCTTGGTGCCATACACAGATACCAG-3′ oligo as forward primer and 5′-GAGCCAACAGGCACCACAGAGAAACCGCAG-3′ oligo as reverse primer, the cDNAs were digested with PvuII to eliminate linker contamination. The amplified cDNAs were concatamerized and cloned into T vector and sequenced at Functional Bioscience. Bioinformatic analysis of individual short RNA clones from the sequencing results has been described previously[51].

### LNA-based miRNA microarray

miRNA profiling in RNAs from naïve, effector and memory T cell were performed using LNA mercury™ microarray at Exiqon (Denmark). The quality of the total RNA was verified by an Agilent 2100 Bioanalyzer profile. A mixture of equal amount of total RNA from naïve, effector and memory T cell were made as reference. 2 µg total RNA from sample and reference pool were labeled with Hy3™ and Hy5™ fluorescent label, respectively, using the miRCURY™ LNA Array labeling kit (Exiqon, Denmark). The Hy3™-labeled samples and a Hy5™-labeled reference pool RNA samples were mixed pair-wise and hybridized to the miRCURY™ LNA array version 8.0 (Exiqon, Denmark), which contains capture probes targeting all human, mouse and rat miRNA listed in the miRBASE version 8.0. The hybridization was performed according to the miRCURY™ LNA array manual using a Tecan HS4800 hybridization station (Tecan, Austria) and the slides were scanned by a ScanArray 4000 XL scanner (Packard Biochip Technologies, USA). Image analysis was carried out using the ImaGene 6.1.0 software (BioDiscovery, Inc., USA). The quantified signals were normalized using the global Lowess (LOcally WEighted Scatterplot Smoothing) regression algorithm.

### 3′RACE RT-PCR based real-time PCR

3′RACE RT-PCR based real-time PCR is modified from previously described methods [52], [53]. Briefly, 300 ng small RNA fraction was polyadenylated with poly(A) tailing kit (Epicentre Biotechnologies, US) according to the manufacturer's instructions. The reaction was extracted with phenol/chloroform, and precipitated with ethanol. The poly(A) tailed small RNAs were annealed with an anchored oligo dT—miR RT oligo dT and transcribed with Superscript III first chain synthesis kit (Invitrogen) according to manufacturer's instruction. cDNAs were amplified with SYBR green 2x mix (Takara) in a icycler system (Bio-rad) with 15 seconds at 95°C and 30 seconds at 60°C for 45 cycles, followed by a thermal denaturing step to generate the melting curves to verify the amplification specificity. Table S5 shows the primers used in this assay.

## Supporting Information

Figure S1 Real time RT-PCR to detect lower frequency miRNAs. Expression of indicated miRNAs in T cell subsets was tested by real time PCR. Expression level was normalized to that of small non-coding RNA U6B. Mean of triplicate experiments±SD is shown.
(0.21 MB TIF)

Table S1 miRNAs cloned from naïve, effector and memory T cells. The cloning frequency of individual microRNAs and their representation as a percentage of total miRNA clones isolated in each T cell subset are shown.
(0.02 MB XLS)

Table S2 Microarray analysis of cloned miRNAs also reveals differential expression in T cell subsets. Hybridization signals obtained in the microarray for miRNAs that were identified by direct cloning are shown.
(0.02 MB XLS)

Table S3 Normalized miRNA cloning frequency in T cell subsets. The percent cloning frequency in Supplemental Table 1 was normalized according to total hybridization signal obtained in the microarray for the corresponding T cell subset to obtain the normalized expression data. Only miRNAs cloned more than 4 times are shown.
(0.02 MB XLS)

Table S4 Sequence of all miRNAs cloned in naïve, effector and memory T cells. Annotated miRNA sequences extracted from the concatemerized small RNA sequencing in different T cell subsets is shown. Only miRNAs cloned more than 4 times are included. The numbers before each sequence represent individual identity marks.
(0.08 MB XLS)

Table S5 Primers used for the 3′RACE-based real time PCR.
(0.01 MB XLS)
