# Supplementary figures and images for: miRNA Profiling of Naïve, Effector and Memory CD8 T Cells
Source: PLoS One. 2007 Oct 10;2(10):e1020. doi: 10.1371/journal.pone.0001020 (PMC2000354; doi:10.1371/journal.pone.0001020)

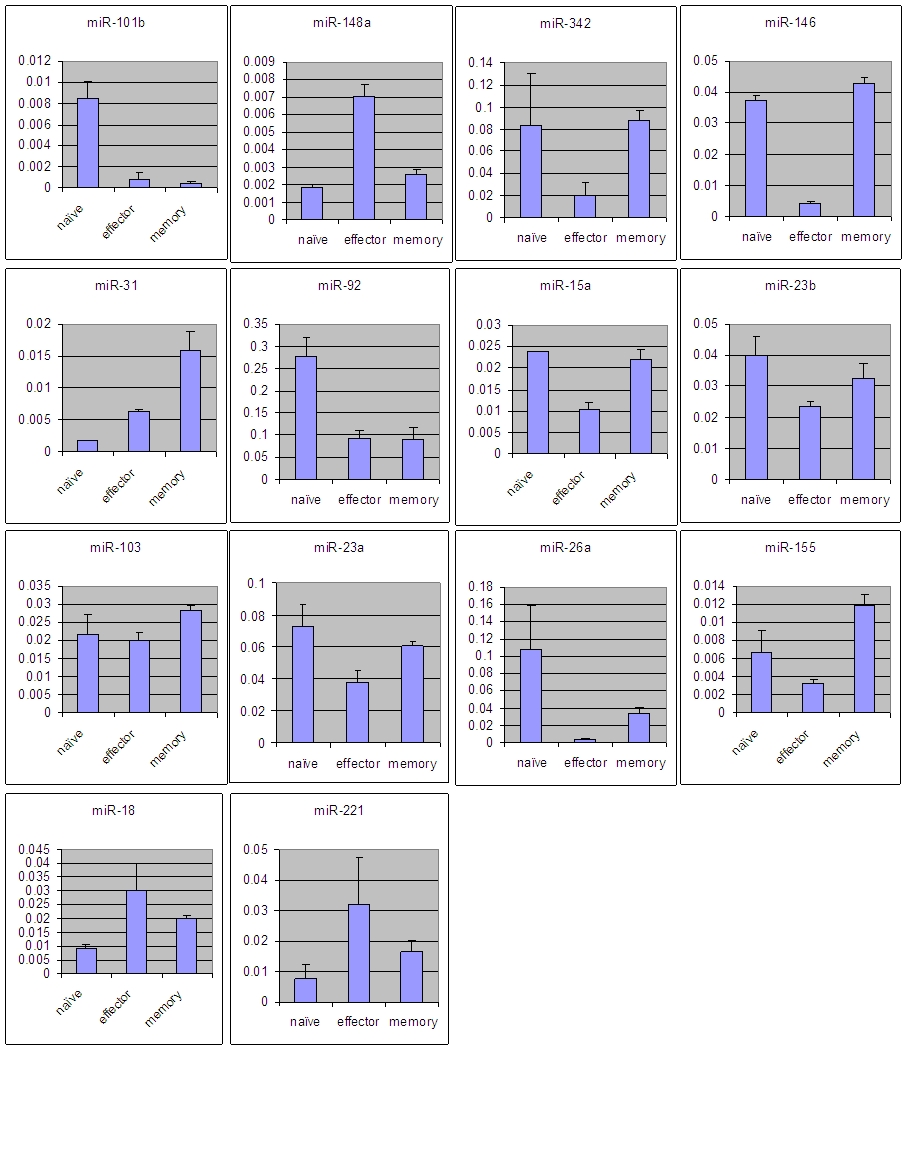

Supplement: Figure S1 — Real time RT-PCR to detect lower frequency miRNAs. Expression of indicated miRNAs in T cell subsets was tested by real time PCR. Expression level was normalized to that of small non-coding RNA U6B. Mean of triplicate experiments±SD is shown. (0.21 MB TIF) [file pone.0001020.s001.tif]
